# Shock Response Characteristics and Equation of State of High-Mass-Fraction Pressed Tungsten Powder/Polytetrafluoroethylene-Based Composites

**DOI:** 10.3390/polym17172309

**Published:** 2025-08-26

**Authors:** Wei Zhu, Weihang Li, Wenbin Li, Xiaoming Wang, Wenjin Yao

**Affiliations:** School of Mechanical Engineering, Nanjing University of Science and Technology, Nanjing 210094, China; 12021059@njust.edu.cn (W.Z.); lwh2020@njust.edu.cn (W.L.); lwb2000cn@njust.edu.cn (W.L.); 202xm@163.com (X.W.)

**Keywords:** shock response, equation of state, tungsten/polytetrafluoroethylene composites, plate impact test, mesoscale simulation

## Abstract

Tungsten powder/polytetrafluoroethylene (W/PTFE) composites have the potential to replace traditional metallic materials as casings for controllable power warheads. Under explosive loading, they generate high-density and relatively uniformly distributed metal powder particles, thereby enhancing close-range impact effects while reducing collateral damage. To characterize the material’s response under impact loading, plate impact tests were conducted to investigate the effects of tungsten content (70 wt%, 80 wt%, and 90 wt%) and tungsten particle size (200 μm, 400 μm, and 600 μm) on the impact behavior of the composites. The free surface velocity histories of the target plates were measured using a 37 mm single-stage light gas gun and a full-fiber laser interferometer (DISAR), enabling the determination of the shock velocity–particle velocity relationship to establish the equation of state. Experimental data show a linear relationship between shock velocity and particle velocity, with the 80 wt% and 90 wt% composites exhibiting similar shock velocities. The fitted slope increases from 2.792 to 2.957 as the tungsten mass fraction rises from 70 wt% to 90 wt%. With particle size increasing from 200 μm to 600 μm, the slope decreases from 3.204 to 2.756, while *c*_0_ increases from 224.7 to 633.3. Comparison of the Hugoniot pressure curves of different specimens indicated that tungsten content significantly affects the impact behavior, whereas variations in tungsten particle size have a negligible influence on the Hugoniot pressure. A high tungsten content with small particle size (e.g., 90 wt% with ~200 μm) improves the overall compressive properties of composite materials. Based on the experimental results, a mesoscale finite element model consistent with the tests was developed. The overall error between the numerical simulations and experimental results was less than 5% under various conditions, thereby validating the accuracy of the model. Numerical simulations revealed the coupling mechanism between tungsten particle plastic deformation and matrix flow. The strong rarefaction unloading effect initiated at the composite’s free surface caused matrix spallation and jetting. Multiple wave systems were generated at the composite–copper interface, whose interference and coupling ultimately resulted in a nearly uniform macroscopic pressure field.

## 1. Introduction

The evolution of modern warfare imposed dual demands on munition systems: high lethality and precise control. Conventional preformed fragment warheads, while effective in causing damage, often pose unavoidable risks of collateral damage. Studies have shown that millimeter-scale fragments propelled by explosions can reach initial velocities of 1500–2000 m/s and maintain residual velocities of 300–500 m/s at a long range [1,2], which pose threats to non-combatants outside the intended damage radius, particularly in asymmetric combat scenarios such as urban counterterrorism and VIP protection. Such operational requirements have driven the rapid development of low-collateral-damage (LCD) munitions. Damage mechanisms based on metal powders attracted significant attention due to their unique momentum-limited characteristics [3,4]. Technological breakthroughs in metal powder-based damage mechanisms originated from a re-evaluation of traditional fragmentation lethality principles. When the size of damage elements is reduced to the sub-millimeter scale, their aerodynamic drag coefficient increases exponentially [5]. This rapid kinetic energy dissipation endows metal powder-based projectiles with significant environmental adaptability: In the near field, high-density powder clouds exhibit collective motion, forming a pressure-like field with considerable momentum transfer efficiency [6]; whereas in the mid-to-far field, the residual kinetic energy of individual particles is significantly reduced. Such dynamically controllable lethality provides a promising technological solution for urban combat scenarios.

Current research on LCD warheads primarily focuses on the dispersal dynamics of explosion-driven particles [5,7,8,9,10,11,12]. Koneru et al. [7] conducted early studies on the jetting behavior of dense particle beds driven by explosive loading, using discrete element method (DEM) collision models to address inter-particle interactions. Kyner et al. [5] investigated the implosion-driven acceleration of spherical particle shells. Loiseau et al. [6] performed experimental studies on shock wave attenuation and particle dispersion in high-energy explosives surrounded by metal/non-metal powder layers. Chen et al. [8] employed multilayer paper targets to investigate the kinetic energy characteristics of metal powder clouds driven by explosives. Bai et al. [9] proposed an improved approach by adding dispersants to metal powders and demonstrated the advantages of segmented dispersal. Li et al. [10] found that incorporating PTFE as a dispersant effectively suppressed tungsten powder sintering. These breakthroughs demonstrate the excellent compatibility between metal/polymer composites and LCD warhead applications.

Current research on the dynamic mechanical behavior of metal/polymer composites is primarily focused on their characterization under quasi-static or low-strain-rate conditions. Several studies investigated the dynamic compression behavior of such materials using impact tests such as the split Hopkinson pressure bar (SHPB) [11,12,13]. Regarding the response mechanisms and experimental techniques of metal/polymer materials under ultra-high strain rates (>10^4^ s^−1^), plate impact and explosive loading tests are commonly employed [14,15,16,17]. Millet [14] conducted shock compression experiments on two alumina–epoxy composites with different compositions to analyze the effect of alumina content on the material’s equation of state and found a clear linear relationship between shock wave velocity and particle velocity. Jordan et al. [15] employed a combination of plate impact and explosive loading techniques to determine the Hugoniot relationships of Al–Fe_2_O_3_–epoxy composites over a wide range of shock pressures. Bober et al. [16] reported a series of plate impact experiments on polymer–metal composites, with metal phase volume fractions ranging from 0% to 40%, and found that the shock response curves were strongly dependent on the composite composition. Rauls et al. [17] investigated the shock compression response of composites consisting of quartz glass spheres embedded in a thermoplastic polymethyl methacrylate (PMMA) matrix using conventional plate impact experiments. Due to limitations in experimental techniques and measurement accuracy, the effects of metallic particle size, mass fraction, and their interaction with the matrix on shock wave propagation characteristics remain inadequately understood.

A growing body of research demonstrated that mesoscale topological structures and particle size significantly affect the dynamic mechanical behavior and shock response of composites [18,19]. Due to the extremely short duration of shock events, the amount of information that can be obtained experimentally is limited, and a comprehensive understanding of the shock structure in such heterogeneous materials requires not only characterization of the macroscopic response, but also analysis of mesoscale features. Numerical simulations offer an effective means of addressing these challenges [20,21,22,23,24,25]. Herbold et al. [20] employed multi-material Eulerian and Arbitrary Lagrangian–Eulerian (ALE) methods to effectively model the dynamic compression behavior of particulate composites under large deformations. Jordan et al. [21] combined equation-of-state experiments with mesoscale simulations to investigate the shock response of epoxy-based particulate composites. Vogler et al. [22] studied the shock wave structure in heterogeneous materials using mesoscale simulations, which explicitly addressed the effects of heterogeneity across multiple length scales. Qiao et al. [23] introduced a mesoscale modeling approach that incorporates realistic morphological distributions and conducted simulations to study the shock compression response of Al–W–binder systems with varying mesoscale configurations. Yang et al. [24] investigated the shock compression behavior of Al/PTFE reactive materials using a single-stage gas gun and developed a 3D mesoscale model based on micro-computed tomography (micro-CT) slice images. Ravindran et al. [25] used two-dimensional finite element modeling to simulate the multiscale shock response of particulate composites consisting of a PMMA matrix and soda–lime glass particles.

This study focuses on tungsten/polytetrafluoroethylene (W/PTFE)-based composites with high tungsten content, which are widely used in controllable lethality warheads but whose shock response remains poorly understood. The effects of varying tungsten mass fractions (70 wt%, 80 wt%, and 90 wt%) and particle sizes (200 μm, 400 μm, and 600 μm) on the shock response characteristics were investigated. Plate impact tests were conducted to capture the free surface velocity histories during shock wave propagation, revealing the Hugoniot relationship of the W/PTFE composites and the dynamic interaction mechanisms between tungsten particles and the PTFE matrix. Furthermore, a two-dimensional mesoscale numerical model was developed based on the experimental setup, and the accuracy of the simulations was validated using experimental results. The finite element (FE) model revealed the deformation and flow behaviors of the two constituent phases under shock loading and provided insights into the internal pressure response of the material. The obtained equations of state and mesoscale modeling approaches for W/PTFE-based composites can be utilized for the design and optimization of controllable warheads.

## 2. Experimental Procedures

### 2.1. Sample Preparation

**Figure 1:**
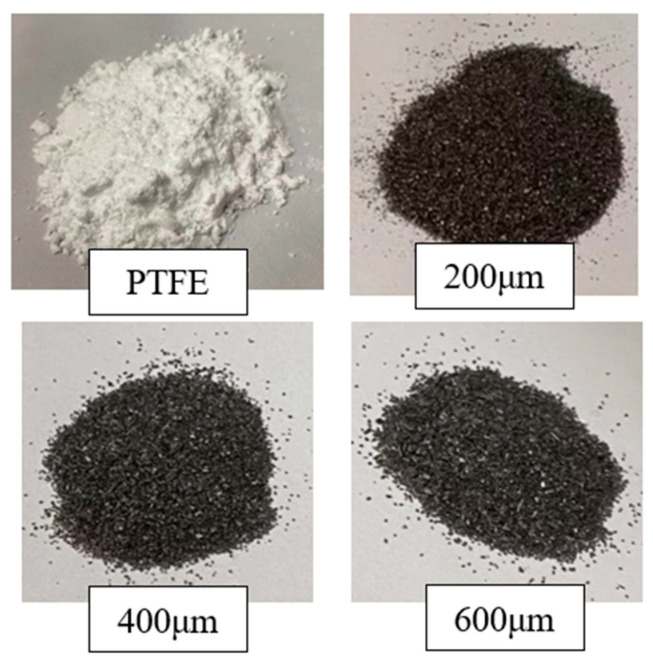
Macroscopic morphology of PTFE resin powder and tungsten powder.

**Figure 2:**
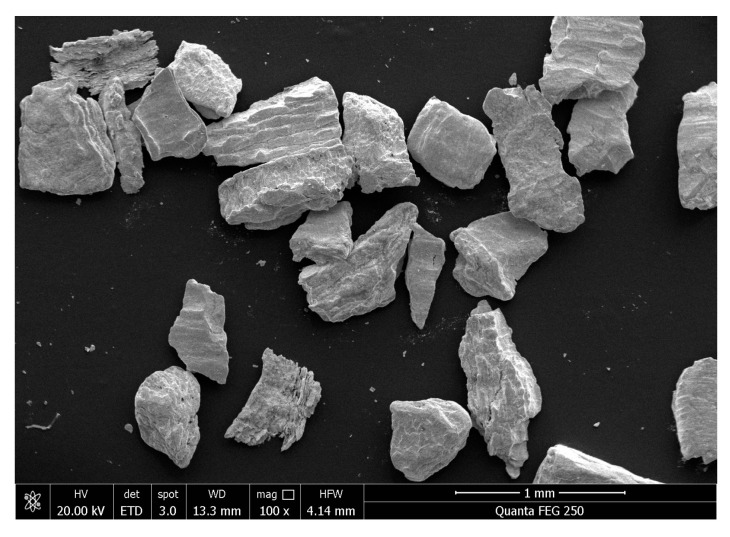
SEM images of 400 μm tungsten particles.

In this study, W/PTFE composite specimens were prepared using 40 μm PTFE powder and pure tungsten powders with particle sizes of 200 μm, 400 μm, and 600 μm. The PTFE powder was supplied by Shenyang Tianyuxiang Micropowder Materials Co., Ltd., Shenyang, Liaoning Province, China, and the tungsten powders were provided by Mudanjiang North Alloy Tool Co., Ltd., Mudanjiang, Heilongjiang Province, China. The morphologies of the raw materials are shown in Figure 1. Scanning electron microscopy (SEM) images of the tungsten particles are presented in Figure 2.

**Figure 3:**
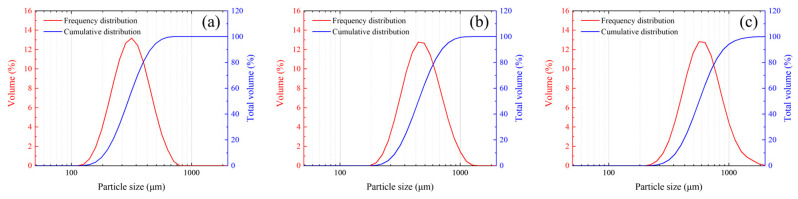
Particle size frequency distribution and cumulative distribution of tungsten particles: (**a**) 200 μm, (**b**) 400 μm, and (**c**) 600 μm. The red curve is the particle frequency distribution curve (left axis) and the blue curve is the particle cumulative distribution curve (right axis).

The frequency and cumulative particle size distributions of the tungsten powders were measured using a Mastersizer 2000 laser particle size analyzer (Malvern Panalytical, Malvern, Worcestershire, UK), as shown in Figure 3. The D50s (median particle sizes) were 237 μm, 422 μm, and 609 μm, respectively. The difference between D90 and D10 was less than 1.5 times the D50, indicating that the particle size span between the 90th and 10th percentiles did not exceed 1.5 times the median size. This demonstrated a relatively uniform particle size distribution, which minimized the presence of extremely coarse or fine particles, and the narrow distribution helped reduce defects during the compaction process.

**Figure 4:**
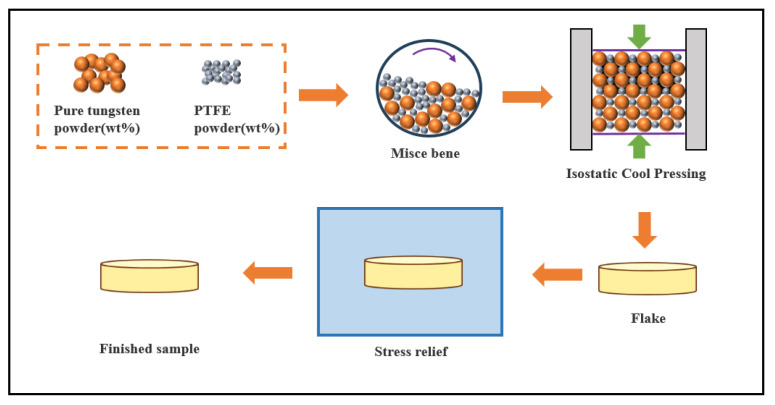
Sample preparation process.

**Figure 5:**
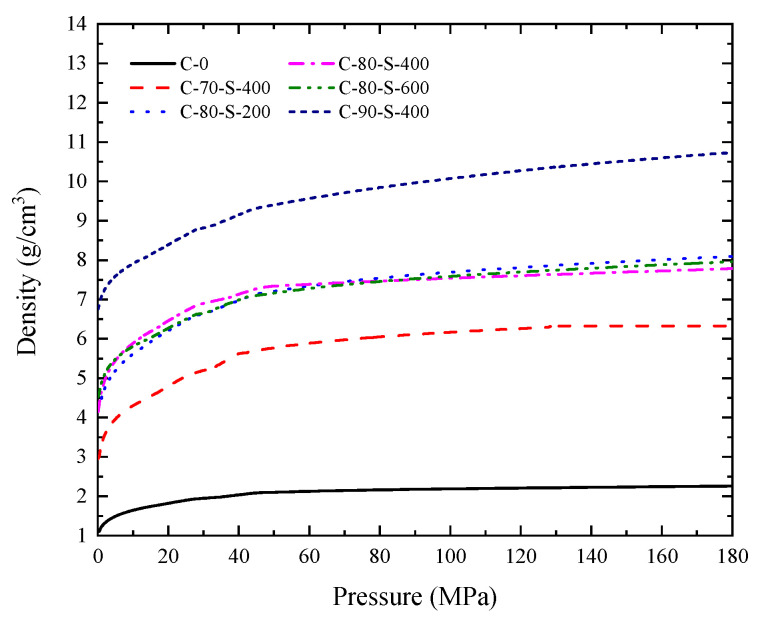
Pressure–density curves of W/PTFE powder compacts under uniaxial pressing.

The preparation process and parameter selection of the specimens were consistent with previous published research [13]. The fabrication process consisted of two stages: mixing and compaction, as illustrated in Figure 4. First, PTFE and tungsten powders were placed in a 3D mixer and tumble-mixed for 15 min to ensure uniform dispersion. The blended powders were then loaded into a mold and subjected to continuous pressure using a uniaxial pressing device at a rate of 0.1 mm/s until the specimen reached the target height. After holding the pressure for 1 min, the compact was demolded, resulting in green bodies with theoretical densities ranging from 5.69 to 10.75 g/cm^3^. To enhance sample consistency, the compacts were left to rest in a thermostatic chamber for 24 h to relieve residual stresses. As shown in Figure 5, the effect of compaction pressure on specimen density exhibits a nonlinear trend, with the rate of density increase leveling off after a critical pressure threshold is exceeded. This process, by optimizing the mixing uniformity and compaction parameters, provided high-density, low-defect specimens suitable for subsequent impact testing.

Samples were designated using the format “C-tungsten content-S-particle size” (e.g., C-80-S-400 represents a sample with 80 wt% tungsten content and 400 μm tungsten particle size). Tungsten contents of the samples included 70 wt%, 80 wt%, and 90 wt%, with corresponding particle sizes of 200 μm, 400 μm, and 600 μm. The density of each sample was measured using the Archimedes method, with three independent tests conducted per sample. Table 1 summarizes the parameters and density variations for samples. The rightmost column in the table represents the density averages, with the overall standard deviation shown in parentheses. The mass deviation of all samples was within ±3%, and the relative densities were ≥95%, confirming the reproducibility of the fabrication process.

### 2.2. Test Equipment Setup

**Figure 6:**
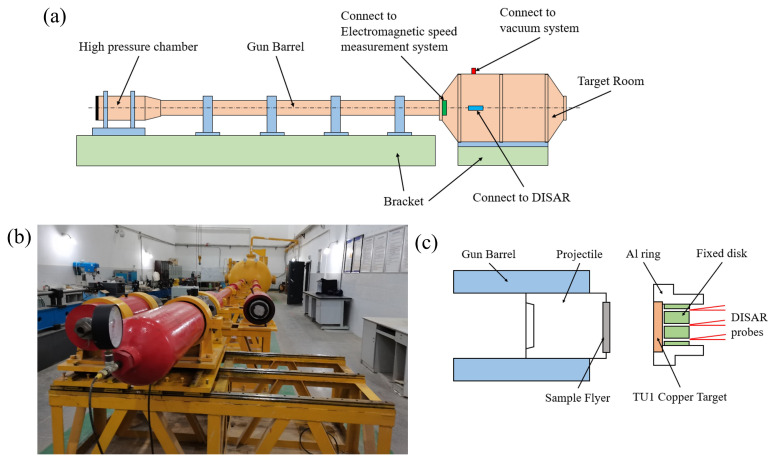
Plate impact experimental setup: (**a**) schematic diagram of the system, (**b**) 37 mm single-stage light gas gun, and (**c**) schematic of the impact zone.

The plate impact test setup is illustrated in Figure 6a,b. The experimental system consists of a loading subsystem and a measurement subsystem. The loading subsystem includes a 37 mm single-stage light gas gun, a vacuum target chamber, a gas driving system, a sabot, and an electromagnetic velocity measurement system. Considering the high diffuse reflectivity of the W/PTFE flyer surface, an asymmetric reverse-collision configuration was adopted. By adjusting the driving pressure and sabot mass, the impact velocity of the W/PTFE composite flyer could be precisely controlled. The plastic response of the copper target was used to calibrate the shock pulse width. Upon impact, a planar shock wave was generated and propagated through the copper target. Due to geometric confinement, the material in the impact zone experienced a quasi-one-dimensional strain state. The impact region is shown in Figure 6c. The measurement subsystem comprises an all-fiber laser interferometric velocimeter (DISAR) and a LeCroy WavePro 760Zi (from Teledyne LeCroy, New York, USA) data acquisition system. This system is employed to measure the displacement or velocity history of moving surfaces [26,27]. The DISAR probe was focused on the free surface of a high-reflectivity copper target, and particle velocity history was extracted via the optical Doppler effect.

### 2.3. Sample Dimension Design

**Figure 7:**
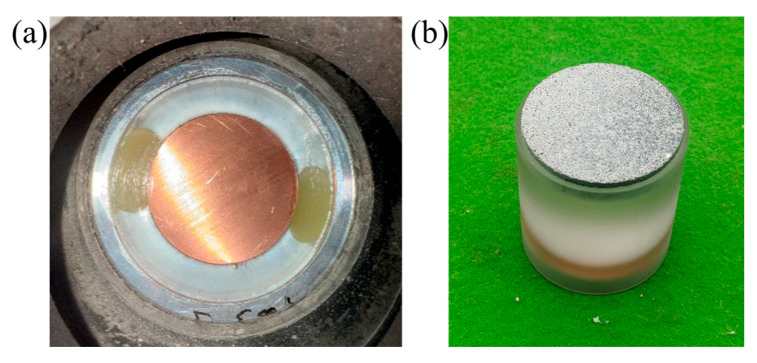
Schematic of the plate impact test setup: (**a**) target plate, (**b**) projectile equipped with sample flyer plate.

To prevent lateral rarefaction waves generated by the flyer and target from interfering with the central impact zone, and to ensure that the shock wave remains a uniform planar front, the flyer and target diameter-to-thickness ratio should exceed 2 [28]. All probes were positioned outside the unloading angle. The designed flyer plates had a thickness of 5.5 mm and a diameter of 35 mm, with end-face parallelism within 0.05 mm and surface flatness within 0.02 mm. The target plates were made of TU1 copper with a density of ρ_1_ = 8930 kg/m^3^, a diameter of 35 mm, and a thickness of 6 mm. The target surfaces were polished prior to testing to remove oxide layers. The target installation is shown in Figure 7a, and the flyer plate assembly with the sabot is illustrated in Figure 7b. To obtain material response data under high-pressure conditions, each flyer specimen was impacted four times at different velocities, ranging from 100 m/s to 900 m/s. For each specific velocity and specimen combination, only one impact test was conducted. A total of 20 valid data points were recorded, corresponding to experiment numbers 001–020.

### 2.4. Data Processing Methods

**Figure 8:**
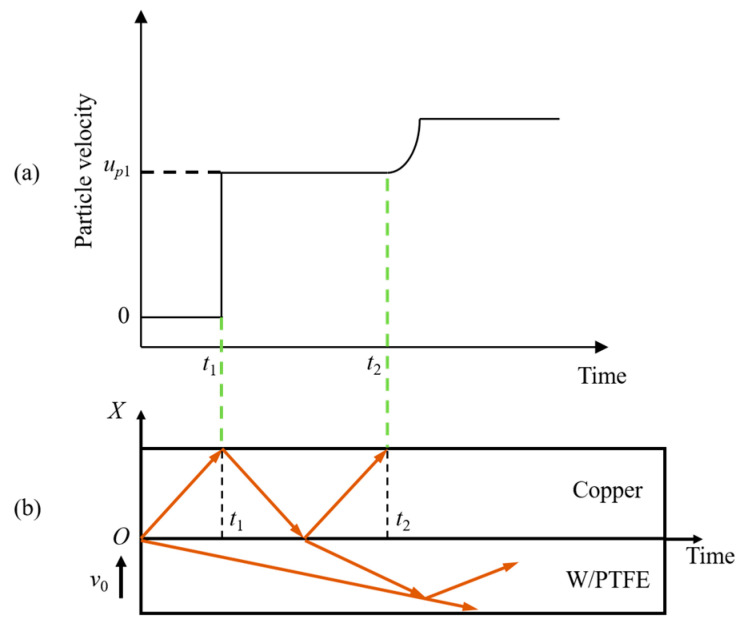
Principle of the plate impact test: (**a**) schematic of particle velocity history at the free surface of the TU1 target plate, (**b**) *X*–*t* diagram illustrating the propagation of the shock wave.

When the flyer impacts the target plate at an initial velocity *v*_0_, a compression wave propagates bilaterally from the impact interface between the flyer and the target. The wave propagation in the *X*–*t* domain is shown in Figure 8b. Due to the high shock wave velocity in TU1 copper, the compression wave reaches the free surface of the target at time *t*_1_, causing a sudden jump in particle velocity at the surface. Simultaneously, a tensile wave is reflected inward from the free surface; this tensile wave then reflects as a compression wave at the impact interface and propagates back to the free surface at time *t*_2_, causing a second particle velocity jump. The probe records a free surface velocity plateau between the arrival times of the two compression waves at the free surface, with an amplitude denoted as *u_p_*_1_, as shown in Figure 8a.

The shock response of a material is commonly described using the intrinsic functional relationship between the shock wave velocity *u_s_* and the particle velocity behind the shock front *u_p_* [29].

(1) $$u_{s}=f(u_{p})$$

Empirically, this functional relationship is often simplified to the following polynomial form:

(2) $$u_{s}=c_{0}+S_{1}u_{p}+S_{2}u_{p}^{2}+\ldots .$$

In this expression, *c*_0_ denotes the sound speed in the material at zero pressure, while *S*_1_ and *S*_2_ are empirical coefficients. Experimental results by Grady [30] and Gebbeken et al. [31,32] indicate that for most materials, when no phase transition occurs, *S*_2_ ≈ 0, and the relationship between shock velocity and particle velocity simplifies to the following:

(3) $$u_{s}=c_{0}+Su_{p}.$$

In this equation, *c*_0_ represents the bulk sound speed at zero pressure, and *S* is the slope of the first derivative of the bulk modulus with respect to pressure. This linear relationship is simple and effectively characterizes the shock response of materials. For TU1 copper, the bulk wave velocity is *c*_1_ = 3940 m/s, and the slope *S*_1_ of the shock velocity–particle velocity (*u_s_*–*u_p_*) relationship is 1.489. The relationship between shock velocity *u_s_*_1_ and particle velocity *u_p_*_1_ for TU1 copper is given by the following:

(4) $$u_{s1}=3940+1.489u_{p1}.$$

The propagation of the shock wave in the TU1 copper target satisfies the Hugoniot jump conditions, from which the Hugoniot stress σ*_H_* in the target can be determined:

(5) $$\sigma_{H}=\rho_{1}c_{1}\left(u_{p1}/2\right)+\rho_{1}S_{1}{\left(u_{p1}/2\right)}^{2}.$$

Due to the continuity at the flyer/target impact interface, the Hugoniot stress σ*_H_* is also applicable to the flyer material. Under one-dimensional strain conditions, the Hugoniot stress σ*_H_* and the hydrostatic pressure *p* are related by the following:

(6) $$\sigma_{H}=p+4\tau_{\max}/3.$$

In the equation, τ_max_ represents the maximum shear stress of the material, which is much smaller than σ*_H_*, and can therefore be neglected. Therefore,

(7) $$p\approx \sigma_{H}.$$

By applying the Hugoniot jump conditions to the specimen, the particle velocity *u_p_*_2_ and shock wave velocity *u_s_*_2_ behind the shock front in the specimen can be determined, respectively.

(8) $$u_{p2}=v_{0}-u_{p1}/2$$

(9) $$u_{s2}=p/\rho_{0}u_{p2}$$

The shock Hugoniot can be obtained by conducting a series of plate impact experiments at different impact velocities and pressures, thereby acquiring multiple data points of shock wave velocity versus particle velocity. These data are then used to establish the *u_s_*–*u_p_* relationship and construct the equation of state.

## 3. Experimental Results and Analysis

### 3.1. Free Surface Particle Velocity

**Figure 9:**
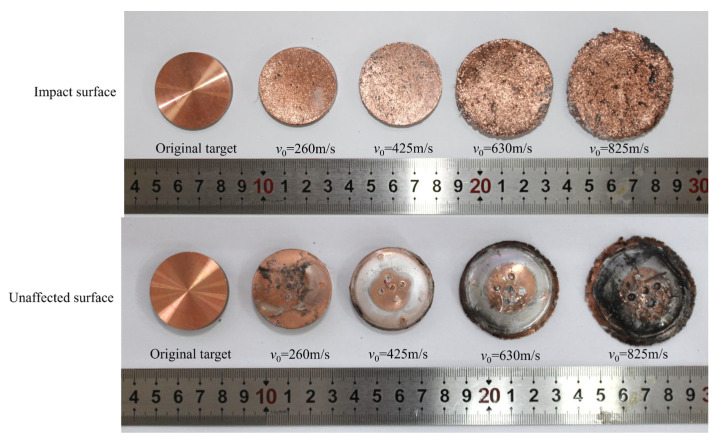
Morphology of the target plates before impact and the target plates after impact at different impact velocities in the C-80-S-400 tests.

Due to the insufficient material strength of the flyer, it disintegrated upon impact, and only the target plate was recovered after the test. Figure 9 shows the target morphologies under different impact velocities for the C-80-S-400 specimen. The target exhibited a central indentation on the impacted surface with a protrusion on the rear side. Small pits caused by tungsten particle penetration were observed on the impacted surface. As the impact velocity increased, the degree of deformation of the target and the penetration depth of the tungsten particles both increased. On the non-impacted surface, several pits corresponding to the positions of the four DISAR probes were observed.

**Figure 10:**
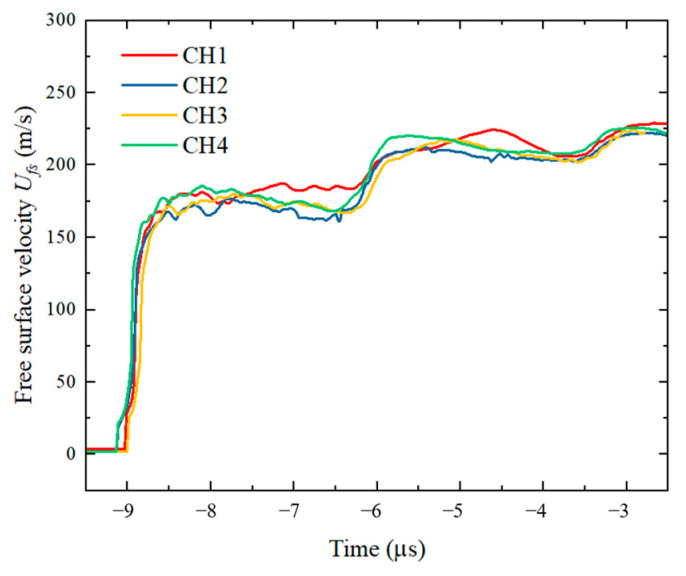
Free surface velocity history of the TU1 target plate impacted by the C-80-S-200 specimen at a velocity of 419 m/s.

**Figure 11:**
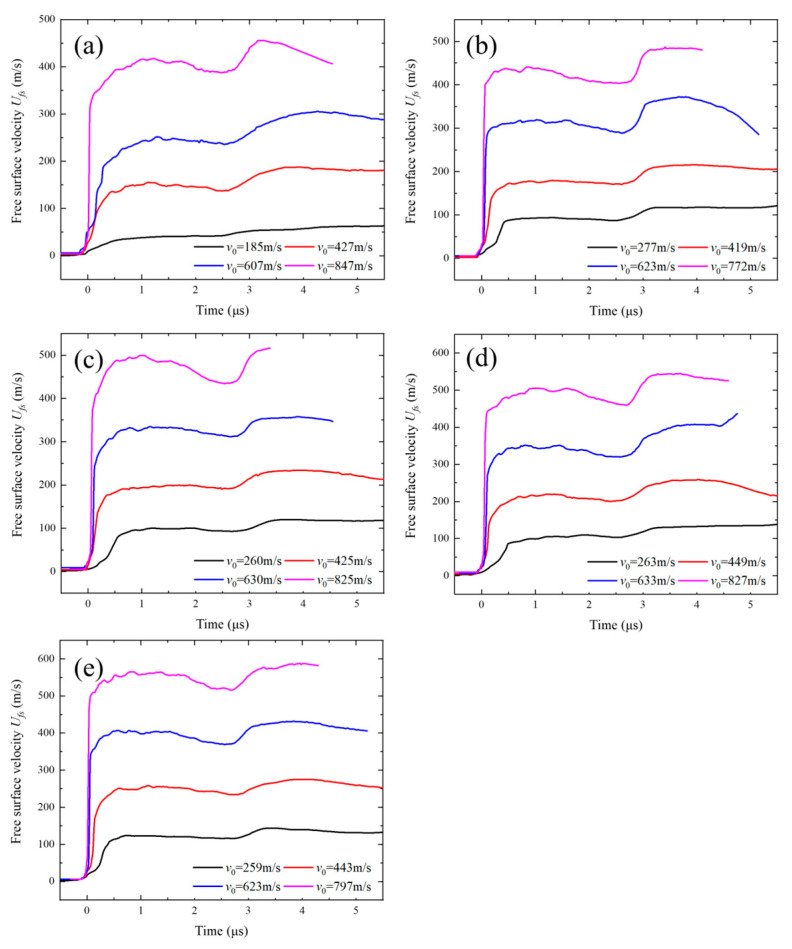
Free surface velocity histories of the TU1 target plate under different impact velocities: (**a**) C-70-S-400, (**b**) C-80-S-200, (**c**) C-80-S-400, (**d**) C-80-S-600, and (**e**) C-90-S-400.

Each experiment produced data from four DISAR probes. To clearly present the shock wave propagation, the free surface velocity from test No. 006, monitored by DISAR, was processed using image algorithms to obtain the particle velocity history at the target free surface, as shown in Figure 10. To eliminate data errors caused by flyer heterogeneity, the average values of the four probe measurements under each experimental condition were calculated, as illustrated in Figure 11a–e. The average particle velocity of the first plateau was taken as *u_p_*_1_. Based on Equations (1)–(7), the Hugoniot stress σ*_H_*, shock wave velocity *u_s_*, and particle velocity *u_p_* were calculated. Table 2 summarizes the results of the plate impact experiment data processing.

### 3.2. Shock Velocity Versus Particle Velocity

For composites containing 70–90 wt% tungsten and particle sizes of 200–600 μm, the shock parameters *c*_0_ and *S* can be linearly fitted as follows:

For C-70-S-400,

*u_s_* = 325.1 m·s^−1^ + 2.792 *u_p_* (10)

For C-80-S-200,

*u_s_* = 224.7 m·s^−1^ + 3.204 *u_p_* (11)

For C-80-S-400,

*u_s_* = 546.5 m·s^−1^ + 2.793 *u_p_* (12)

For C-80-S-600,

*u_s_* = 633.3 m·s^−1^ + 2.756 *u_p_* (13)

For C-90-S-400,

*u_s_* = 435.0 m·s^−1^ + 2.957 *u_p._* (14)

**Figure 12:**
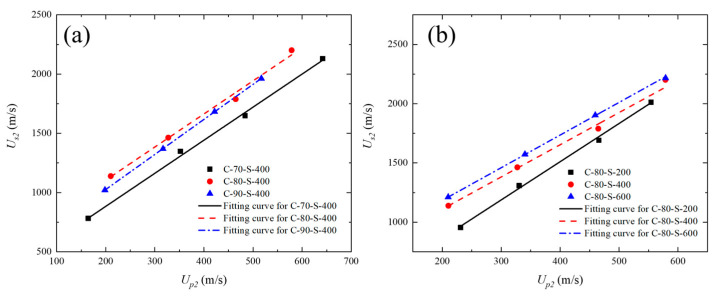
Relationship between shock wave velocity and particle velocity (*u_s_*–*u_p_*) for W/PTFE composites and the corresponding fitting curves: (**a**) effects of different tungsten mass fractions; (**b**) effects of different tungsten particle sizes.

As shown in Figure 12a, the fitted curves for C-80-S-400 and C-90-S-400 are highly similar. Therefore, a unified fitting was performed for both using Equation (3):

*U_s_* = 478.1 m·s^−1^ + 2.901 *u_p._* (15)

**Figure 13:**
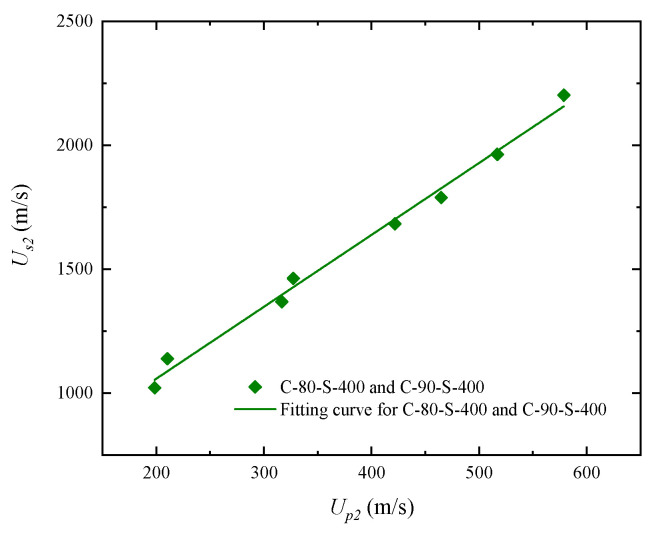
Unified linear fitting curve of shock wave velocity versus particle velocity (*u_s_*–*u_p_*) for C-80-S-400 and C-90-S-400 composites.

The linear fitting results for the two materials are shown in Figure 13.

As shown in the Hugoniot relationship curves (*u_s_*–*u_p_*) in Figure 12a, the fitted slope *S* increases from 2.792 to 2.957 as the tungsten mass fraction rises from 70 wt% to 90 wt%. Specifically, increasing the tungsten fraction reduces the proportion of the ductile PTFE matrix and decreases the inter-particle spacing, thereby limiting the overall plastic deformation capability and increasing the impact stiffness. Moreover, the high-density and brittle nature of tungsten particles intensifies stress concentrations at the phase interfaces, promoting microcrack initiation and energy dissipation under high-velocity impact, which further compromises the composite’s compressive strength. Figure 12b presents the fitted *u_s_*–*u_p_* curves for materials with different tungsten particle sizes. With particle size increasing from 200 μm to 600 μm, the slope S decreases from 3.204 to 2.756, while *c*_0_ increases from 224.7 to 633.3. As tungsten particle size increases, the interface density decreases substantially. Larger tungsten particles result in more direct wave propagation paths with reduced reflection and energy dissipation, thereby increasing the macroscopic wave speed of the material. However, due to their inability to pack densely, the local porosity also rises with particle size, making the composite more prone to damage modes, such as pore collapse and compaction under shock loading, ultimately reducing its effective compressive resistance. Furthermore, larger particles possess smaller and weaker interfacial bonding areas with the matrix, increasing the likelihood of interfacial sliding and debonding during dynamic loading. Overall, a high tungsten content with small particle size (e.g., 90 wt% with ~200 μm) improves the overall compressive properties of composite materials.

**Figure 14:**
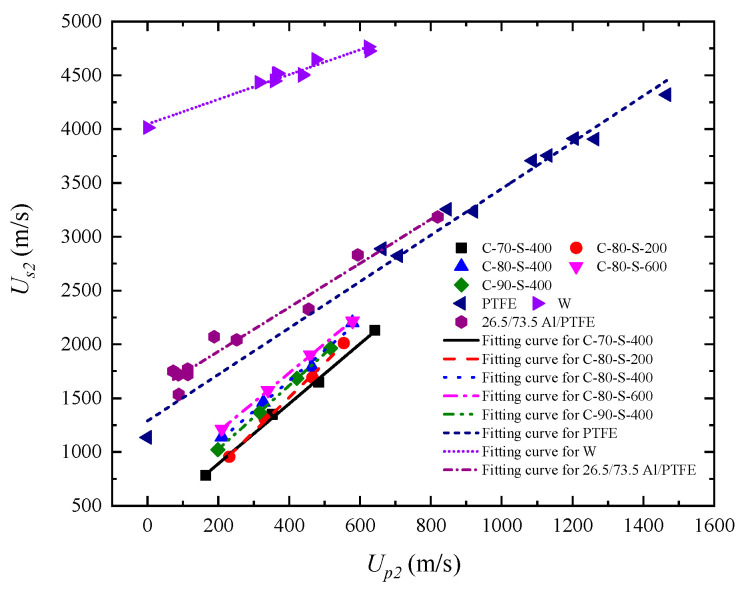
Comparison of the shock wave velocity–particle velocity (*u_s_*–*u_p_*) relationships between the composites in this study and those of Al/PTFE composites [24], tungsten (W) [33], and PTFE [33].

Furthermore, a comparison of Hugoniot characteristics for different metal/polymer systems is shown in Figure 14. An Al/PTFE composite with a mass ratio of 26.5/73.5 was prepared by powder mixing, drying, cold isostatic pressing, and vacuum sintering. The mixture consisted of aluminum powder with a particle size of 75 to 150 μm and a purity of 99% and PTFE powder with a particle size of 150 μm [24]. The product had an average density of 2.235 g/cm^3^. It can react and release energy under shock loading while remaining inert in ambient conditions. It is observed that the *u_s_*–*u_p_* curve of the low-density Al/PTFE composite (Al 26.5 wt%) closely resembles that of pure PTFE. This similarity can be attributed to the relatively small density difference between aluminum (2.7 g/cm^3^) and PTFE (~2.2 g/cm^3^), as well as the low filler content in the 26.5 wt% Al/PTFE composite, which is insufficient to dominate the composite’s overall response. Additionally, aluminum exhibits relatively good interfacial compatibility with PTFE, forming a compliant transition zone that facilitates energy transfer and absorption. As a result, low-density, low-modulus metal particles have a limited effect on the dynamic response of the composite at low filler contents, with the matrix remaining the dominant phase. In contrast, W/PTFE composites with high tungsten content show a significantly higher *S* value and a lower *c_0_* value compared to PTFE, indicating that tungsten particles substantially alter the shock response, following a mechanism distinct from that of low-density metals. Tungsten has a much higher density (19.3 g/cm^3^), which leads to pronounced wave impedance mismatch within the material, causing repeated reflection and refraction of shock waves. Moreover, the mechanical properties of tungsten differ greatly from those of PTFE. At high mass fractions, tungsten particles become the dominant phase in the composite, governing its overall response under shock loading. This contrast highlights that the density, modulus, and interfacial compatibility of metal particles with the matrix collectively determine the dynamic response behavior of metal/polymer composites under extreme loading conditions.

### 3.3. Shock Stress Versus Particle Velocity

**Figure 15:**
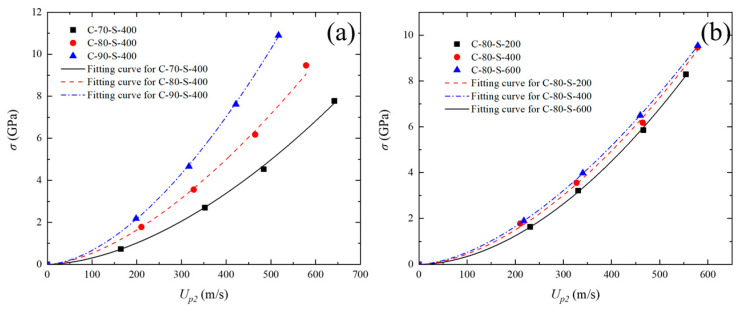
Relationship between shock stress and particle velocity (σ*_H_*–*u_p_*) for W/PTFE composites and corresponding fitting curves: (**a**) comparison among C-70-S-400, C-80-S-400, and C-90-S-400; (**b**) comparison among C-80-S-200, C-80-S-400, and C-80-S-600.

**Figure 16:**
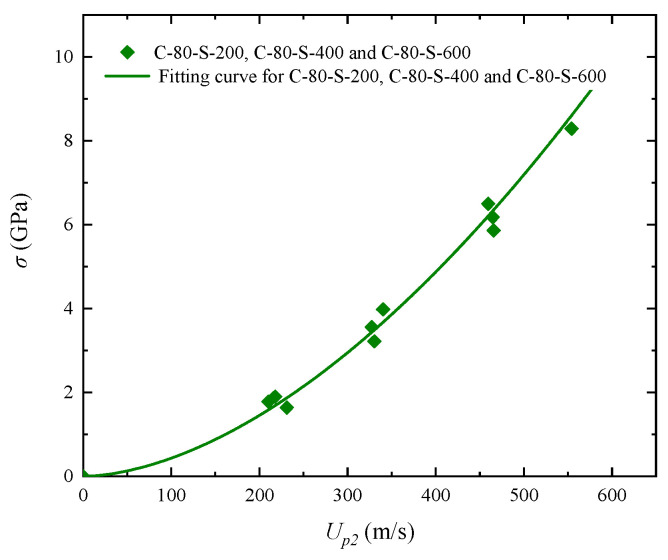
Unified fitting curve of shock stress versus particle velocity (σ*_H_*–*u_p_*) for C-80-S-200, C-80-S-400, and C-80-S-600 W/PTFE composites.

Figure 15a,b presents the Hugoniot shock pressure–particle velocity (σ*_H_*–*u_p_*) relationships for the composites under shock stresses ranging from 0.73 to 10.90 GPa. The curves were obtained by fitting the measured *c*_0_ and *S* parameters for five different materials. As shown in Figure 15a, the compressibility of the W/PTFE composites decreases with increasing tungsten content. Under the same shock stress, the C-90-S-400 specimen exhibits the lowest particle velocity, indicating the strongest resistance to compression. In the later stage of shock compression, the σ*_H_*–*u_p_* curves exhibit highly similar trends in stress increase. Although the *u_s_*–*u_p_* relationships of C-80-S-400 and C-90-S-400 are nearly identical, their σ*_H_*–*u_p_* curves differ significantly due to the variation in material density under shock compression. This density enhancement effect is one of the main driving factors for the altered shock response in high-tungsten composites. Figure 15b further compares the σ*_H_*–*u_p_* curves of composites with the same tungsten content but different particle sizes. The results show that particle size has a negligible effect on the shock pressure–particle velocity relationship, as the three curves are nearly overlapping. The corresponding fitted results are shown in Figure 16, indicating that under high-pressure shock conditions, the pressure response becomes significantly less sensitive to particle size. This phenomenon may be attributed to damage mechanisms, such as interfacial debonding and particle fragmentation, dominating the energy dissipation process under extreme loading, thereby weakening the geometric effect of the initial particle size.

**Figure 17:**
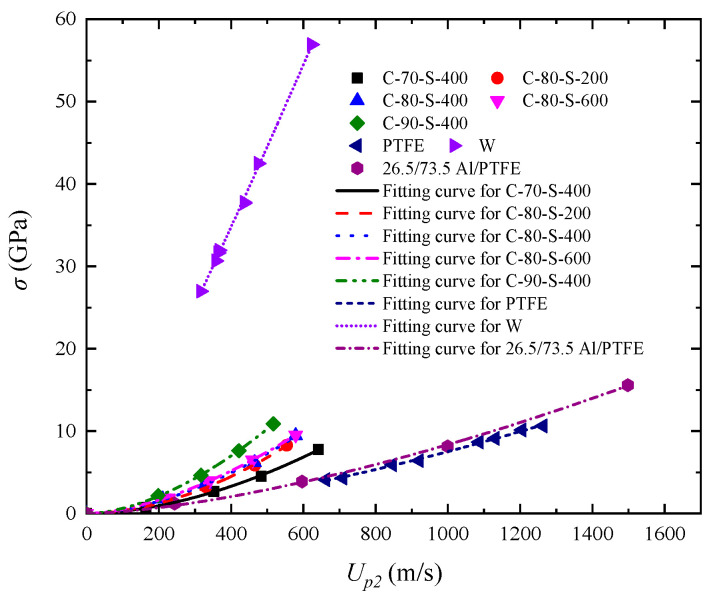
Comparison of the σ*_H_*–*u_p_* relationships obtained in this study with those of Al/PTFE [24], tungsten (W) [33], and PTFE [33].

Figure 17 compares the Hugoniot pressure curves of PTFE, pure tungsten, and the Al/PTFE systems report. The results show that the σ*_H_*–*u_p_* curves of all W/PTFE specimens lie between those of pure tungsten and PTFE. Notably, the stress increase trend in the high-pressure region is closer to that of PTFE, which sharply contrasts with the behavior of low-aluminum-content Al/PTFE systems reported by Yang et al. [24]. The high modulus and high density of tungsten particles significantly enhance the dynamic stiffness of the composite through increased inertial effects, while the visco-plastic flow of the PTFE matrix dominates the stress relaxation process at high pressures, causing the composite to retain polymer-like characteristics in the later stages of shock loading.

It is worth emphasizing that in this study, when calculating particle velocity using Equation (3), the Hugoniot stress σ*_H_* is assumed to be equivalent to the hydrostatic pressure *p*, neglecting the influence of shear strength τ, and thereby treating the composite as a fluid-like medium according to Equation (4). For the tungsten/PTFE composites, the PTFE matrix mainly consists of viscoelastic molecular chains that exhibit certain flowability under shock compression. Therefore, the effect of shear strength was ignored in analyzing the shock performance. Overall, under shock loading, the density and volumetric strain of W/PTFE increase with pressure. Microscopically, the inter-molecular or atomic distances decrease under high pressure, resulting in minute volumetric compression at the micro scale. Macroscopically, W/PTFE behaves as a brittle material due to the random distribution of tungsten particles; particle clusters undergo stress concentration and shear fracture under complex stress states, leading to significant changes in density and volumetric strain.

## 4. Mesoscale Numerical Simulation

### 4.1. Random Particle Generation

In this study, tungsten particles were generated randomly on a two-dimensional plane. Prior to particle placement, the model geometry was meshed based on its predefined shape and dimensions. Using the generated mesh information, all node and element data of the component were recorded, the total area of the component was calculated, and the geometric boundaries along with the node numbers located on these boundaries were identified.

**Figure 18:**
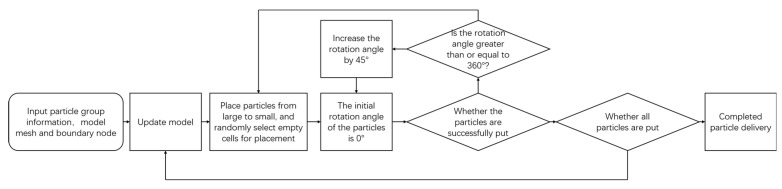
Flowchart of random particle generation process.

The generation process begins with creating a single irregularly shaped particle. In the polar coordinate system, the position of any point *P_i_* in the two-dimensional plane is determined by the parameters *r* and *θ*. The vertex coordinates are calculated as shown in Equation (15), and then converted into Cartesian coordinates. The particle area is computed by summing the areas of constituent triangles. The complete procedure for particle generation is illustrated in Figure 18.

(16) $$\left\{\begin{array}{l} r=r_{i}\times \left[1+random(-1,1)\times f_{r}\right]\\ \theta =2\pi /\alpha \times \left[\beta +random(-1,1)\times f_{\theta }\right] \end{array}\right.$$

In the equation, *r_i_* represents the random radius of an arbitrary point *P_i_*, which is controlled based on the predefined particle size limits. The parameter *f_r_* denotes the radial fluctuation ratio (ranging from 0 to 1); a larger value corresponds to a more irregular particle shape. Similarly, *f*_θ_ represents the angular fluctuation ratio (ranging from 0 to 0.5), and increasing its value also leads to greater irregularity in particle geometry. The parameter α defines the number of particle sides, while β denotes the number of particle vertices. The function random (−1, 1) generates a random number within the range (−1, 1), introducing stochastic perturbations to the particle geometry.

Particles are sequentially inserted in descending order of diameter. The mesh elements of the target component are classified as deposited and undeployed. The center of each particle is placed at a randomly selected undeployed element. A particle is considered successfully inserted if none of its internal elements intersect boundary elements or the external boundary nodes. Upon successful insertion, the undeployed element list is updated by removing the internal elements occupied by the new particle. This process is repeated iteratively, generating new particles until the total particle area reaches the specified volume fraction. Figure 18 illustrates the random particle placement algorithm. The resulting mesostructured model consists of element sets for particles, matrix, and interfaces, to which corresponding material properties can be directly assigned.

### 4.2. Numerical Simulation Model

**Figure 19:**
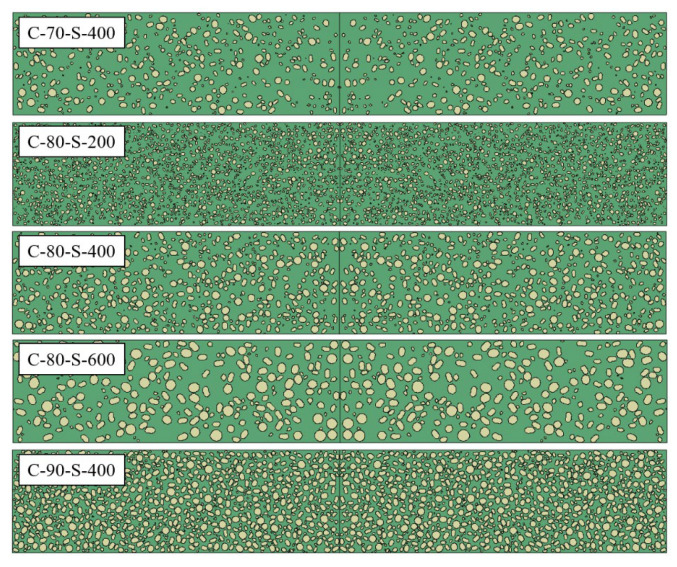
Two-dimensional mesoscale model of W/PTFE composite material constructed based on particle placement (green: PTFE matrix; yellow: tungsten particles).

**Figure 20:**
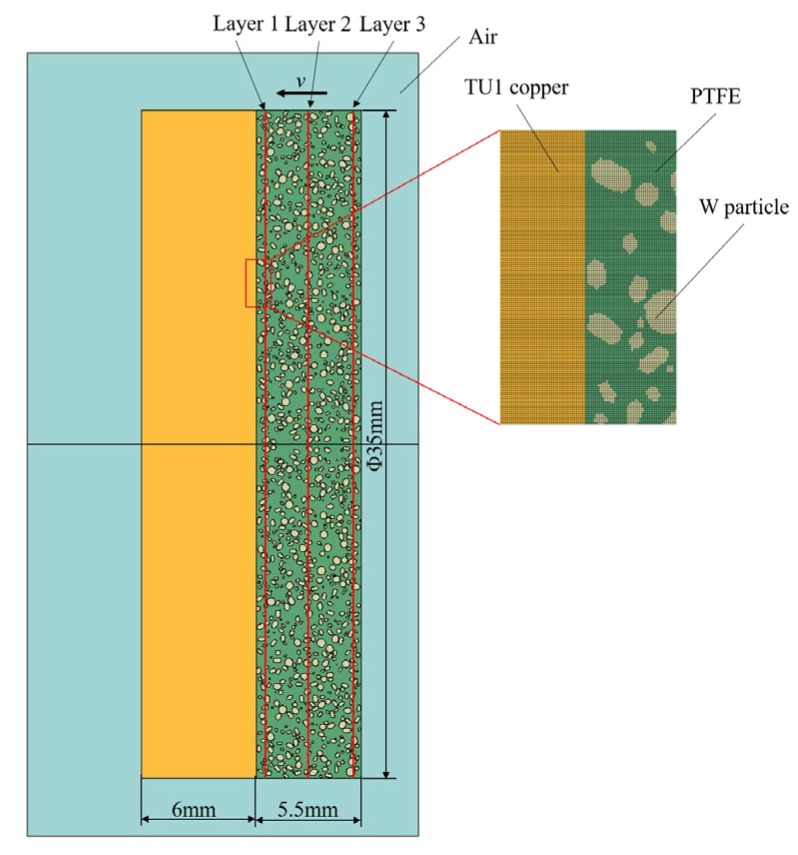
Complete 2D finite element model.

In this study, a two-dimensional axisymmetric mesoscale model was developed using the commercial finite element software LS-DYNA R11 (developed by LSTC, Livermore, CA, USA, now part of Ansys Inc., Canonsburg, PA, USA) to numerically simulate the impact of W/PTFE composite flyers on TU1 copper targets. The model measures 20 mm × 40 mm. The geometric parameters of the model were strictly aligned with the experimental setup: both the flyer and the target had diameters of 35 mm, with thicknesses of 5.5 mm and 6 mm, respectively. As shown in Figure 19, the mesoscale flyer model was constructed using a stochastic particle placement method to reproduce the spatial distribution of tungsten particles within the PTFE matrix. The tungsten volume fraction was consistent with that of the experimental specimens. The flyer and target are surrounded by air. The multi-material Eulerian formulation was employed to handle large deformation processes. A multi-material Eulerian formulation is a computational method that simulates the dynamic behavior of multiple interacting materials on a fixed grid. It excels at modeling extreme deformation, mixing, and fragmentation by tracking material volumes within cells and solving shared conservation laws. Material interface evolution was tracked by calculating the Eulerian volume fraction (EVF) within each element, ensuring high fidelity in interface representation. The air domain edges are set as non-reflecting boundaries. The flyer is simulated with an initial velocity consistent with the initial conditions of the test. The Euler mesh size is 0.02 mm, and the model uses a uniform quadrilateral mesh with a mesh number of 1,000,000. The simulation was conducted using the cm-g-μs unit system. Along the impact direction of the flyer, three layers of monitoring elements were deployed: the first and third layers were located 0.25 mm from the material boundaries, while the middle layer was positioned equidistantly between them with an interlayer spacing of 2.5 mm. The physical quantities of each layer were obtained by statistically averaging the values over the corresponding element groups, as shown in Figure 20.

### 4.3. Material Model

During impact loading, materials are subjected to high pressure and large deformations. The constitutive modeling of mechanical behavior under dynamic loading typically consists of two components: a strength model that governs the yield stress, and an equation of state (EOS) that defines the hydrostatic pressure as a function of density and internal energy [23,34]. In this study, the Johnson–Cook model was adopted for both tungsten particles and the PTFE matrix. This model is widely used for materials subjected to large strains, high strain rates, and elevated temperatures, as it describes material strength as a function of plastic strain, strain rate, and temperature. The von Mises equivalent flow stress is defined as follows [35]:

(17) $$\sigma =\left(A+B\varepsilon^{n}\right)\left(1+C\ln\varepsilon^{*}\right)\left\{1-{\left[\left(T-T_{room}\right)/\left(T_{melt}-T_{room}\right)\right]}^{m}\right\}$$

where *A*, *B*, *C*, *n*, and *m* are material-specific constants. ε represents the equivalent plastic strain, and ε^∗^ is the dimensionless plastic strain rate. *T* is the current temperature, while *T_room_* and *T_melt_* denote the room temperature and the melting point of the material, respectively. The model parameters used in this study are summarized in Table 3.

In LS-DYNA, the Mie–Grüneisen equation of state is employed to define the pressure *P* of compressed materials:

(18) $$P=\rho_{0}c_{0}^{2}\eta \left(1-\gamma_{0}\eta /2\right)/{\left(1-s\eta \right)}^{2}+\gamma_{0}\rho_{0}E$$

where *c*_0_, *s*, and γ_0_ are material constants, η = ρ/ρ_0_ − 1, and *E* denotes the specific internal energy. The parameters used in the equation of state are listed in Table 4.

### 4.4. Numerical Simulation Results and Verification

**Figure 21:**
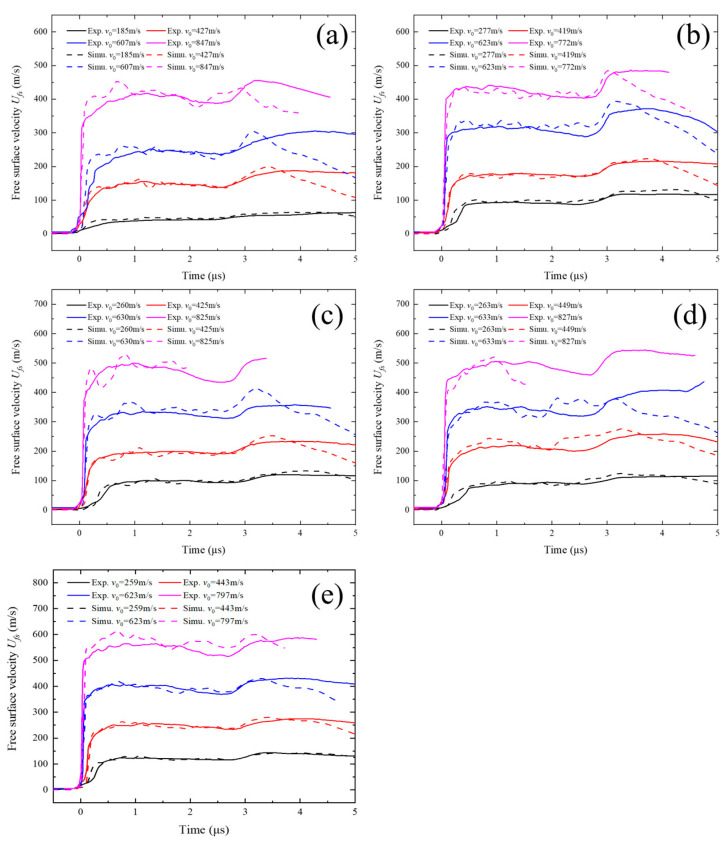
Comparison of particle velocity between numerical simulation and experimental results for samples (**a**) C-70-S-400, (**b**) C-80-S-200, (**c**) C-80-S-400, (**d**) C-80-S-600, and (**e**) C-90-S-400.

The free surface particle velocity response was characterized by extracting the velocity of the central element at the rear surface of the TU1 target plate. Figure 21 presents a comparison between the particle velocities obtained from numerical simulations and plate impact tests under different tungsten particle sizes and volume fractions. Under identical impact velocities, the average relative error between simulation and experimental results ranges from −0.66% to 4.96%, with the overall error remaining within 5%, as shown in Table 5. This level of agreement indicates good consistency between simulation and experiment, effectively validating the predictive accuracy of the finite element model developed in this study. The observed data scatter can be primarily attributed to the following factors: (1) discrepancies between the numerical model and the actual particle size distribution and morphology in the composite; and (2) simplifications in modeling the internal voids within the material. These inherent differences account for the slight deviations between simulated and experimental results.

## 5. Discussion

### 5.1. Particle Deformation and Matrix Flow

**Figure 22:**
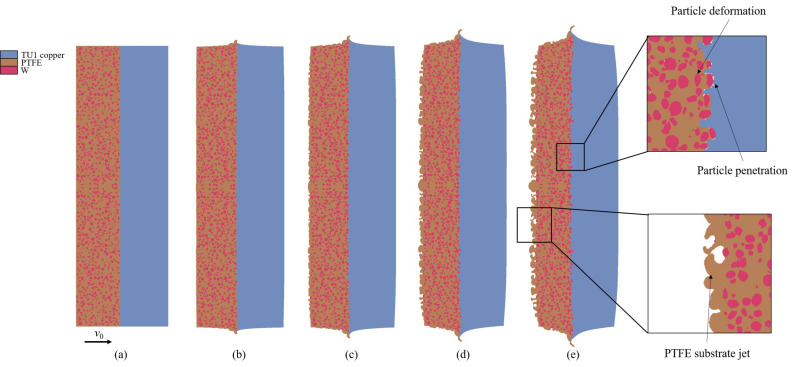
Original state and states after impact at different velocities for C-80-S-400 at *t* = 2.6 μs: (**a**) initial state, (**b**) *v*_0_ = 260 m/s, (**c**) *v*_0_ = 425 m/s, (**d**) *v*_0_ = 630 m/s, and (**e**) *v*_0_ = 825 m/s.

Upon impact, tungsten particles embedded in the PTFE matrix undergo compression and deformation under the influence of the shock wave. Figure 22 illustrates the material’s initial configuration and its deformed states under different impact velocities at the same time point (*t* = 2.6 μs). Compression first occurs in the impact interface region, initiating plastic deformation of tungsten particles along the shock direction. Under high pressure, the PTFE matrix exhibits visco-plastic flow, which may lead to interfacial separation between the matrix and the particles. Meanwhile, the high-impedance tungsten particles act as rigid obstacles, forcing the matrix to deflect significantly around them. The high-modulus tungsten particles also penetrate into the copper target. It is noteworthy that due to the lateral free boundary conditions in the model, lateral rarefaction occurs when the shock wave reaches the boundary, resulting in severe deformation near the edges. These phenomena are consistent with the results of the copper target recovered after the experiment. As the impact velocity increases, the propagation speed of the shock wave also increases, causing the wave front to reach the free surface of the flyer earlier. This leads to an intensified rarefaction unloading effect, which enhances material spallation and jetting behavior, ultimately resulting in an increased number of micro-jets.

**Figure 23:**
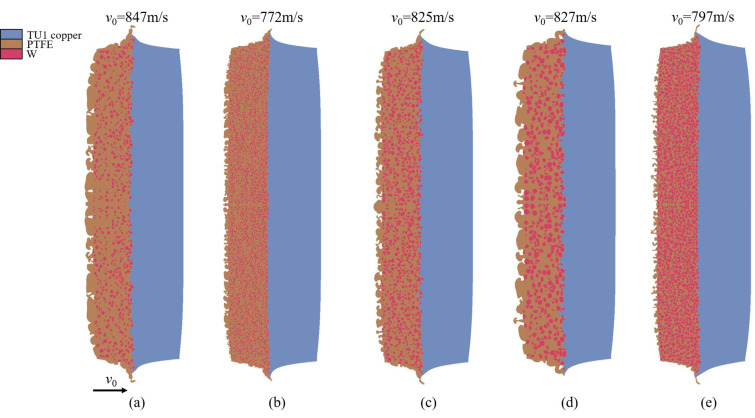
States of W/PTFE composites with different tungsten content and particle sizes under impact velocities of 722 m/s to 847 m/s at *t* = 2.6 μs: (**a**) C-70-S-400, (**b**) C-80-S-200, (**c**) C-80-S-400, (**d**) C-80-S-600, and (**e**) C-90-S-400.

Figure 23 presents a comparative analysis of the impact responses of W/PTFE composites with varying tungsten contents (70–80 wt%) and particle sizes (200–600 μm) at the same time instant within an impact velocity range of 722–847 m/s. It is observed that with constant tungsten content, increasing the particle size leads to a reduction in the degree of particle plastic deformation. Simultaneously, the penetration depth of particles increases, the diameter of impact craters expands, and the crater distribution density decreases. As the particle size grows, the average diameter of micro-jets formed by PTFE matrix spallation on the rear surface increases, whereas the number of micro-jets decreases. Under a fixed particle size, increasing the tungsten content results in a higher density of impact craters, while the penetration depth and crater diameter remain stable. Correspondingly, the micro-jets exhibit a typical pattern of reduced size but a significant increase in quantity. These phenomena can be attributed to two factors: enhanced uniformity in particle distribution improves the mesoscale homogeneity of the material, and the reduction in PTFE matrix volume fraction effectively suppresses the amount of micro-jet ejection.

### 5.2. Analysis of Shock Wave Propagation Characteristics

**Figure 24:**
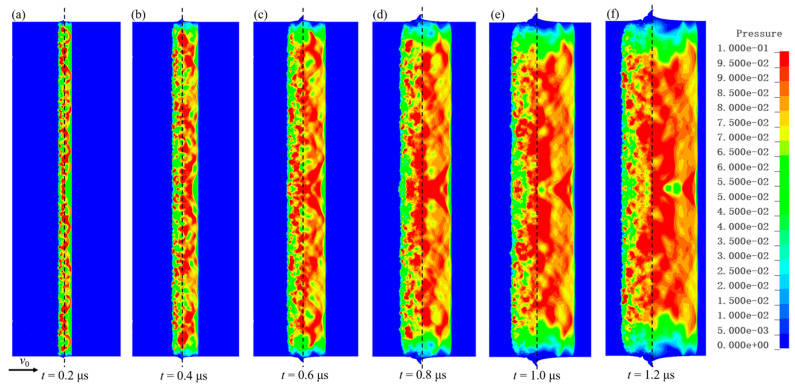
Pressure contour maps of C-80-S-400 at an impact velocity of 825 m/s at different times: (**a**) 0.2 μs, (**b**) 0.4 μs, (**c**) 0.6 μs, (**d**) 0.8 μs, (**e**) 1.0 μs, and (**f**) 1.2 μs.

Figure 24 presents pressure contour maps of the C-80-S-400 composite at various time points under an impact velocity of 825 m/s. The shock wave propagates from the impact interface toward the free surfaces on both sides, separating the W/PTFE composite and the copper target into uncompressed and compressed regions. Compared to its propagation in the copper target, the shock wave travels relatively slower within the composite. When comparing different impact velocities, the shock pressure increases with rising impact velocity. Due to the heterogeneity of the composite, post-shock pressures inside the material exhibit nonuniformity. Multiple intermittent impacts from the PTFE matrix and tungsten particles induce alternating shock interactions at different locations on the copper target interface. The repeated convergence and coupling of shock waves generate multisource wave systems at the copper interface whose interference and coupling macroscopically form a quasi-uniform pressure field.

**Figure 25:**
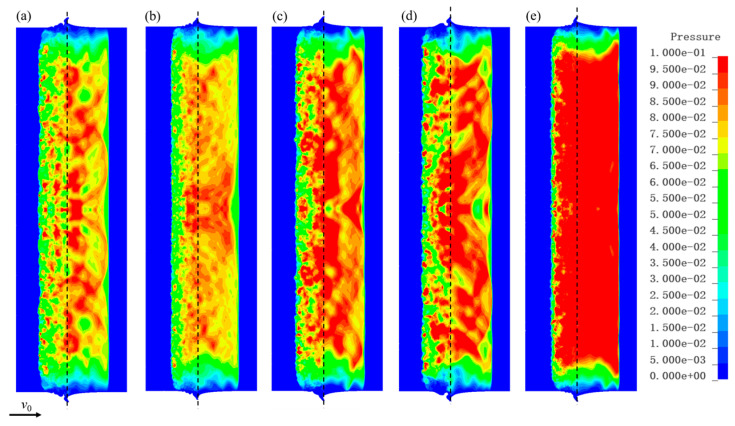
Pressure contour maps at *t* = 1 μs for W/PTFE composites with different tungsten contents and particle sizes under impact velocities of 722 m/s to 847 m/s: (**a**) C-70-S-400, (**b**) C-80-S-200, (**c**) C-80-S-400, (**d**) C-80-S-600, and (**e**) C-90-S-400.

Figure 25 further reveals the pressure distribution characteristics at the same time instant (*t* = 1 μs) for composites with varying tungsten contents (70–80 wt%) and particle sizes (200–600 μm) subjected to impact velocities ranging from 722 m/s to 847 m/s. The peak pressure shows a positive correlation with tungsten content, while pressure fluctuations are smaller when particle size increases to 400–600 μm. This phenomenon indicates that the dynamic response of the composite is significantly more sensitive to component content than particle size, being primarily governed by impedance matching controlled by the tungsten phase volume fraction.

**Figure 26:**
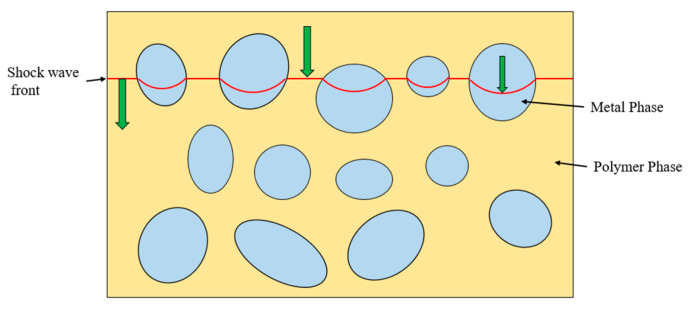
Schematic illustration of shock wave fronts inside metal/polymer matrix composites.

The impact response of two-phase materials under shock loading can be qualitatively explained by shock impedance theory, as illustrated in Figure 26. When a strong shock wave transmits from one material to another, the interface pressure and particle velocity depend on the impedance characteristics of the materials. Tungsten possesses a higher impedance compared to PTFE. During the transmission of a shock wave from a low-impedance material to a high-impedance material, the particle velocity decreases, the shock wave velocity increases, and the pressure rises. Due to the greater inertia of the high-impedance material, particle motion encounters stronger resistance, resulting in reduced transmitted particle velocity. The conservation of momentum at the interface requires pressure continuity, and the high impedance necessitates a reduction in particle velocity to maintain pressure equilibrium. Meanwhile, part of the energy is reflected back into the original medium as a reflected shock wave, causing an increase in interface pressure.

**Figure 27:**
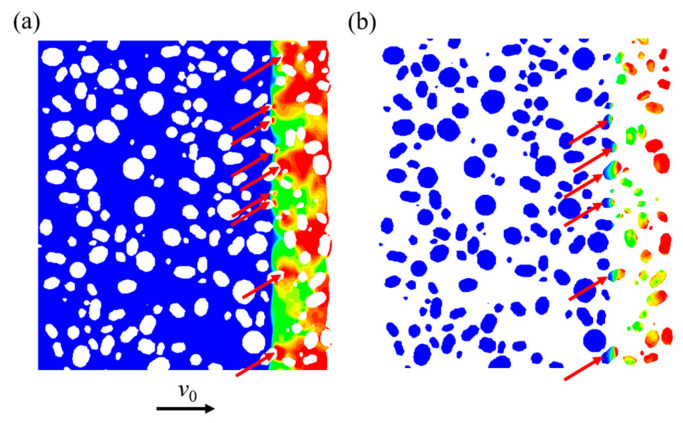
Distinct heterogeneous features of the material’s dynamic response at *t* = 0.2 μs: (**a**) localized high-pressure zones in the matrix; (**b**) gradient distribution of shock wave pressure within particles. The red arrows indicate the locations of local high-pressure areas.

When the shock wave passes through the PTFE-W interface, the material exhibits significant heterogeneity and multiscale coupling in its dynamic response, as shown in Figure 27. The shock wave velocity rapidly increases within the tungsten particles, while the particle velocity sharply decreases. During this process, the pressure is significantly amplified, resulting in localized high-pressure zones. Reflection waves at the interface generate rarefaction waves within the PTFE, which reduces the subsequent shock pressure. Simultaneously, the geometric heterogeneity of the tungsten particles induces wavefront distortion, leading to a pressure gradient distribution of the shock wave.

### 5.3. Hugoniot Pressure Evolution Characteristics

**Figure 28:**
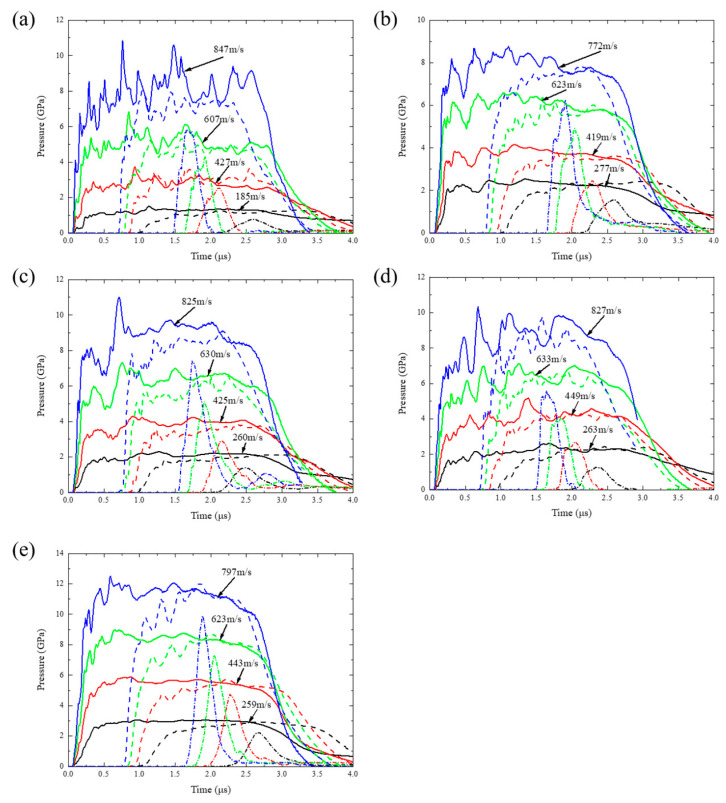
Simulated Hugoniot pressure results. Solid lines represent the average values of the first layer elements, dashed lines represent the average values of the second layer elements, and dash-dot lines represent the average values of the third layer elements: (**a**) C-70-S-400, (**b**) C-80-S-200, (**c**) C-80-S-400, (**d**) C-80-S-600, and (**e**) C-90-S-400.

Figure 28 presents the pressure evolution curves at three monitoring layers, revealing multiscale energy dissipation mechanisms. For instance, at impact velocities ranging from 722 m/s to 847 m/s, the near-field layer (L1) exhibits a pressure plateau averaging 7.93–11.47 GPa and lasting 2.26–2.45 μs, with oscillation amplitudes positively correlated to particle size. The mid-field layer (L2) shows a decay in the pressure plateau to 7.05–10.88 GPa. In the far-field layer (L3), no distinct pressure plateau forms, and peak pressure further decreases to 5.59–9.89 GPa. At the mesoscale, small particle size and high tungsten content enhance stress homogenization by increasing interface density. At the macroscale, the interplay between rarefaction waves and residual stresses governs the evolution of pressure gradients. Notably, near the free surface, the underdeveloped pressure field undergoes rapid unloading due to rarefaction waves, resulting in the disappearance of the pressure plateau.

## 6. Conclusions

This study employed plate impact loading techniques to investigate the dynamic compressive response of tungsten powder/poly(tetrafluoroethylene) (W/PTFE) composites with varying tungsten mass fractions (70 wt%, 80 wt%, and 90 wt%) and tungsten particle sizes (200 μm, 400 μm, and 600 μm). Additionally, a two-dimensional mesoscale finite element model was established and validated against experimental data. Using this numerical model, the deformation behavior of tungsten particles under impact compression and the flow characteristics of the PTFE matrix were further explored. Based on these results, the Hugoniot pressure response characteristics of high-content, pressed W/PTFE composites were analyzed. The main conclusions are as follows:

(1) The impact characteristics of the composites in terms of shock wave velocity, particle velocity, and impact stress were obtained. Experimental results show a linear relationship between shock wave velocity and particle velocity, with the C-80-S-400 and C-90-S-400 samples exhibiting very similar trends, which were fitted uniformly. Increasing tungsten content reduces ductility, thereby limiting overall plastic deformation capacity and increasing impact stiffness. As tungsten particle size increases, interface density significantly decreases. Larger tungsten particles reduce energy dissipation, resulting in an increase in the macroscopic wave speed of the material. Impact stress exhibits a nonlinear increase with particle velocity, while particle size variation has minimal influence on the pressure–particle velocity relationship. Compared to existing Al/PTFE composites (Al 26.5 wt%), tungsten particles dominate the composite response at high mass fractions, controlling the overall dynamic behavior under impact. This comparison highlights that the density, modulus, and interface compatibility of metal particles collectively determine the dynamic response of metal/polymer composites under extreme loading.
(2) A full-scale two-dimensional finite element model was developed, wherein tungsten particles were randomly distributed within the PTFE matrix, consistent with experimental volume fractions, employing a multi-material Eulerian algorithm to handle severe deformation. Simulation results demonstrate good agreement between the particle velocity at the free surface of the target plate and experimental measurements, with the average relative error across conditions ranging from –0.66% to 4.96%, maintaining an overall error within 5%. Data dispersion primarily arises from discrepancies between the numerical model and actual composites in terms of particle size distribution and morphology, as well as simplifications regarding internal porosity effects in the finite element modeling.
(3) Numerical analysis revealed the deformation of tungsten particles and the flow behavior of the matrix under impact compression. The impact interface region experienced initial compression, inducing plastic deformation of tungsten particles along the impact direction, with some particles penetrating into the copper target, which is consistent with experimental observations. The strong rarefaction unloading effect at the composite free surface caused matrix delamination and jetting. Moreover, alternating impacts between the PTFE matrix and tungsten particles generated multiple wave systems at the copper target interface whose interference coupled macroscopically to form a near-uniform pressure field. Near the impact end, the pressure exhibited a plateau, while internal material heterogeneity caused oscillations. At the mesoscale, small particle size and high tungsten content enhanced stress homogenization by increasing interface density; at the macroscale, the interaction between rarefaction waves and residual stresses dominated the evolution of pressure gradients.

## Figures and Tables

**Table 1:** Flyer plate specimen configurations.

No.	Specimens	Tungsten Mass Fraction %	Tungsten Volume Fraction %	Particle Size (μm)	Ideal Density (g/cm^3^)	Average Measured Density (g/cm^3^)
1	C-70-S-400	70	20	400	5.69	5.57 (±0.109)
2	C-80-S-200	80	30	200	7.44	7.32 (±0.098)
3	C-80-S-400	80	30	400	7.44	7.29 (±0.102)
4	C-80-S-600	80	30	600	7.44	7.35 (±0.134)
5	C-90-S-400	90	50	400	10.75	10.66 (±0.101)

**Table 2:** Plate impact test results of tungsten powder/polytetrafluoroethylene composites.

No.	Specimens	*v*_0_ (m/s)	*U_fs_* (m/s)	*U_p_*_2_ (m/s)	*U_s_*_2_ (m/s)	σ*_H_* (GPa)
001	C-70-S-400	185	41.3	164.4	782.6	0.73
002		427	149.3	352.3	1348.1	2.70
003		607	246.3	483.8	1648.3	4.53
004		847	410.3	641.9	2131.0	7.78
005	C-80-S-200	277	91.7	231.1	955.2	1.64
006		419	176.9	330.5	1308.8	3.22
007		623	314.3	465.8	1691.4	5.86
008		772	435.4	554.3	2012.0	8.29
009	C-80-S-400	260	99.4	210.3	1138.7	1.78
010		425	195.3	327.4	1463.1	3.56
011		630	330.7	464.7	1788.9	6.18
012		825	492.5	578.7	2201.4	9.47
013	C-80-S-600	263	105.6	210.2	1212.6	1.90
014		449	217.4	340.3	1573.4	3.98
015		633	346.8	459.6	1902.8	6.50
016		827	496.0	579.0	2217.1	9.54
017	C-90-S-400	259	121.1	198.4	1022.0	2.18
018		443	252.7	316.7	1369.0	4.66
019		623	402.6	421.7	1682.8	7.62
020		797	560.2	516.9	1963.1	10.90

**Table 3:** Parameters of strength models of the materials.

Material	*A* (MPa)	*B* (MPa)	*n*	*C*	*m*	*T_melt_* (K)
Tungsten [36]	1200	1030	0.19	0.034	0.4	1723
PTFE [18]	11	44	0.120	1.00	1.00	650
TU1 Copper [35]	90	292	0.31	0.025	1.09	1356

**Table 4:** Equation of state parameters of the material.

Material	ρ_0_ (g/cm^3^)	*c*_0_ (m/s)	*s*	γ_0_
Tungsten [33]	8.93	4040	1.23	1.80
PTFE [33]	2.152	1841	1.707	0.59
TU1 Copper [33]	8.92	3940	1.45	2.04

**Table 5:** Comparison of experimental and numerical simulation results.

No.	Specimens	*v*_0_ (m/s)	Experimental *U_fs_* (m/s)	Numerical Simulation *U_fs_* (m/s)	Error %	Average Error %
001	C-70-S-400	185	41.3	47.1	14.04	4.96
002		427	149.3	150.8	1.00	
003		607	246.3	248.6	0.93	
004		847	410.3	426.1	3.85	
005	C-80-S-200	277	91.7	94.4	2.94	1.39
006		419	176.9	174.7	−1.24	
007		623	314.3	333.2	6.01	
008		772	435.4	426.0	−2.16	
009	C-80-S-400	260	99.4	95.5	−3.92	−0.66
010		425	195.3	190.1	−2.66	
011		630	330.7	345.4	4.45	
012		825	492.5	490.1	−0.49	
013	C-80-S-600	263	90.4	96.7	6.97	3.21
014		449	217.4	230.2	5.89	
015		633	346.8	353.3	1.87	
016		827	496.0	486.7	−1.88	
017	C-90-S-400	259	121.1	117.3	−3.14	−0.21
018		443	252.7	248.1	−1.82	
019		623	402.6	407.9	1.32	
020		797	560.2	575.8	2.78	

## Data Availability

The authors will supply the relevant data in response to reasonable requests.
